# Cross-pollination of research findings, although uncommon, may accelerate discovery of human disease genes

**DOI:** 10.1186/1471-2350-13-114

**Published:** 2012-11-28

**Authors:** Marlena Duda, Tristan Nelson, Dennis P Wall

**Affiliations:** 1 The Center for Biomedical Informatics, Harvard Medical School, Boston, MA, USA; 2 Department of Pathology, Beth Israel Deaconess Medical Center, Boston, MA, USA

## Abstract

**Background:**

Technological leaps in genome sequencing have resulted in a surge in discovery of human disease genes. These discoveries have led to increased clarity on the molecular pathology of disease and have also demonstrated considerable overlap in the genetic roots of human diseases. In light of this large genetic overlap, we tested whether cross-disease research approaches lead to faster, more impactful discoveries.

**Methods:**

We leveraged several gene-disease association databases to calculate a Mutual Citation Score (MCS) for 10,853 pairs of genetically related diseases to measure the frequency of cross-citation between research fields. To assess the importance of cooperative research, we computed an Individual Disease Cooperation Score (ICS) and the average publication rate for each disease.

**Results:**

For all disease pairs with one gene in common, we found that the degree of genetic overlap was a poor predictor of cooperation (r^2^=0.3198) and that the vast majority of disease pairs (89.56%) never cited previous discoveries of the same gene in a different disease, irrespective of the level of genetic similarity between the diseases. A fraction (0.25%) of the pairs demonstrated cross-citation in greater than 5% of their published genetic discoveries and 0.037% cross-referenced discoveries more than 10% of the time. We found strong positive correlations between ICS and publication rate (r^2^=0.7931), and an even stronger correlation between the publication rate and the number of cross-referenced diseases (r^2^=0.8585). These results suggested that cross-disease research may have the potential to yield novel discoveries at a faster pace than singular disease research.

**Conclusions:**

Our findings suggest that the frequency of cross-disease study is low despite the high level of genetic similarity among many human diseases, and that collaborative methods may accelerate and increase the impact of new genetic discoveries. Until we have a better understanding of the taxonomy of human diseases, cross-disease research approaches should become the rule rather than the exception.

## Background

The pace of genetic discovery in human diseases has accelerated exponentially through the invention of high-throughput technologies and the advent of next-generation sequencing approaches. What were once considered to be well-defined boundaries between human diseases are increasingly appearing as arbitrary and blurred. Recent research has shown that many human diseases share large numbers of genes and genetic networks, and therefore likely share molecular mechanisms that will elucidate shared causes and shared treatments [1].

This emerging picture of commonality among human diseases suggests that research, in particular our collective understanding of the genetic causes of human diseases, should benefit from a shift away from singularly focused research towards multi-disease focused efforts that look across existing disease circumscriptions rather than within. Indeed, recent efforts have begun to prove out this hypothesis, including comparative studies among autism and related neurodevelopmental disorders [2], as well as various disease-network approaches [3]. Such innovative approaches to the study of human diseases will facilitate the creation of a more appropriate genetically based taxonomy of disease.

In the present study, we capitalized on the last fifteen years of genetic research to address whether or not cross-pollination in genetic research of human diseases has served to increase the pace of discovery. Specifically, we constructed authoritative gene lists for 193 human diseases defined in the Medical Subject Headings database [4] and mapped the publication records for each disease to the citation histories of all others. This enabled us to track patterns of shared discovery and evaluate the impact on the rate of new gene discovery in cases where cross-disease research occurred, as well as in cases where it did not.

## Methods

### The Mutual Citation Score

In order to quantify the degree of association between the studies of biologically correlated disorders, we developed the Mutual Citation Score (MCS). The MCS reflects the degree of relation between the genetic research fields of two given diseases (*i*, *j*) based on the frequency with which one disease field cites the work of the other, and is defined by the following equation.

(1) $$MCS_{\mathrm{ij}}=\frac{C_{\mathrm{ij}}+C_{\mathrm{ji}}}{P_{i}+P_{j}}$$

C_*ij*_= Number of citation events where a gene discovery in disease *i* is cited back to the discovery of the same gene in disease *j*

C_*ji*_= Number of citation events where a gene discovery in disease *j* is cited back to the discovery of the same gene in disease *i*

P_*i*_= Total number of genetic publications in *i*

P_*j*_= Total number of genetic publications in *j*

We attempted to control for self-citation by excluding citation events where both publications had the same last author. We computed the MCS for 10,853 disease pairs that had at least one gene in common. Fundamentally, for a pair of diseases, the MCS describes the percentage of all genetic discoveries that cross-reference the other disease. Theoretically, the values for the MCS scale from 0–1, where 0 signifies no record of cross-citations between the two diseases. However, because the MCS is based on the event when a gene is initially discovered in a disease, it would be impossible to have a disease pair with an MCS=1. For all 193 diseases, the research tool Genotator [5] was used to provide gene lists and genetic publication histories dated from 1996–2010 (Genotator files obtained March 2011). Unrelated publications were omitted from the publication histories of stroke, asthma, hypersensitivity, leukemia, diabetes mellitus and hemochromatosis after manual examination of the abstracts revealed no mention of the disease. Publication dates as well as author and citation information were obtained via PubMed Entrez Utilities [6]. Although citation histories were only provided for PubMed Central publications, this subset of 3868 articles supplied an accurate representation of general research trends between diseases. No disease was significantly under or overrepresented in PubMed Central (Additional file 1: Table S1) and the disorders were all treated equally using this systematic approach.

### The Individual Disease Cooperation Score

To determine the level of participation of any distinct disease field in cooperative research, we calculated the Individual Disease Cooperation Score (ICS) for each of the 193 diseases studied. The ICS is a score that reflects the amount of cooperative research that occurred in any individual disease field (*i*) relative to all other diseases, and is defined by the following equation:

(2) $$ICS_{i}=\sum MCS_{\mathrm{ij}}$$

In other words, the ICS for any disease (*i*) is simply the summation of all MCS_(*i*,*j*)_ values for which (*i*) is part of the disease pair (*i*, *j*).

## Results

### Genetic similarity does not predict cooperative research

**Figure 1:**
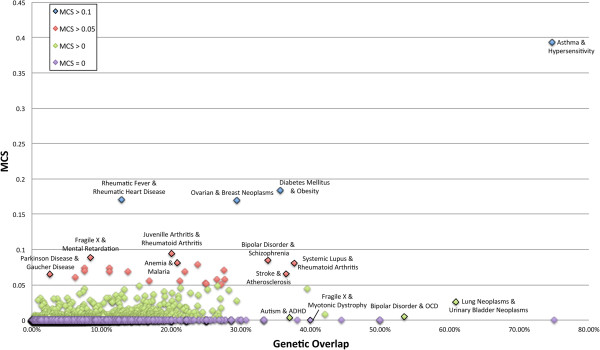
**Mutual Citation Score of all disease pairs as compared with the biological association between the diseases (genetic overlap).** From this figure we can conclude that degree of cooperative research between a disease pair is not always indicative of the intensity of their genetic relationship (r^2^=0.3187).

We calculated the Mutual Citation Score (MCS), a quantification of the degree to which one disease field monitors the genetic discovery of another biologically comparable disease. For 10,853 pairs of genetically related diseases the overall genetic similarity between two disorders was found not to be a consistent predictor of MCS (r^2^=0.3198) (Figure 1). Of all the disease pairs studied for which there is an overlap of at least one gene, over 99% cross-cited their identical genetic findings less than 5% of the time (MCS < 0.05) and 89.56% never cross-referenced their identical genetic findings (MCS = 0) (Additional file 2: Table S2).

The disease pair of asthma and hypersensitivity, two highly genetically related and co-morbid conditions [7], returned the highest MCS of all disease pairs (MCS=0.39297), translating to mutual citation in approximately 40% of all publications relevant to either disorder. This pair shared 455 (74.71%) of their total combined genes, and 1066 citation events were recorded for 59 (13%) of the genes common to both disorders. Among the cross-referenced genes were several known to be high priority candidate genes in both asthma and hypersensitivity, including GSTP1 [8], ADAM33 [9] and ADRB2 [10].

Diabetes mellitus and obesity [11] had the second highest MCS of 0.18314. The genetic overlap (35.7%) between this pair consisted of 418 genes. We found that 118 (28%) of the genes shared between diabetes mellitus and obesity were cross-referenced between the two research fields, including many genes that are strongly linked to both diseases such as FTO [12], involved in regulation of global metabolic rate and body fat accumulation, as well as ADIPOQ [13], which is implicated in the control of fat metabolism and insulin sensitivity.

We observed that a vast majority of disease pairs (99.75%) returned MCS values lower than 0.05, irrespective of the amount of genetic overlap between the pair. For example, lung neoplasms and bladder neoplasms were found to share 431 genes (60.88%), the third highest genetic overlap of all disease pairs examined. Less than 5% (n=21) of the genes common to both diseases were cross-referenced, contributing to the comparatively low MCS of 0.02578. Despite the low level of cooperative research, many of the genes in common, including GSTM1 [14] and TP53 [15], have been shown to be highly associated with the molecular pathology of both diseases.

A similar pattern was observed for bipolar disorder and obsessive-compulsive disorder, two diseases not only known to be genetically linked but also known to occur together commonly in patients [16,17]. We found that this disease pair had a genetic overlap of 53.48% consisting of 307 genes, however the only cross-disease citations were for BDNF [18] and SLC6A4 [19], genes that are widely implicated in over 300 other disorders and potentially less directly related to the mechanistic causes of disease than other genes. The resulting MCS for the pair was 0.004732, thus mutual citation between the fields of bipolar disorder and obsessive-compulsive disorder occurred in less than 0.5% of their combined set of genetic publications.

Table 1 highlights the large variation of Mutual Citation Scores for disease pairs with comparable genetic overlaps. For example, the pair Parkinson disease and Gaucher disease had a genetic overlap of 2.57% and returned an MCS of 0.064803, a very high MCS relative to other disease pairs with a similar genetic overlap such as fragile X syndrome and attention deficit disorder with hyperactivity (ADHD) (MCS=0.002577) as well as α-1 antitrypsin deficiency and lung neoplasms (MCS=0.000996). Also, many diseases that had considerably higher levels of genetic overlap than the Parkinson-Gaucher disease pair had much lower MCS values, such as autism and ADHD (37.00% overlap, MCS=0.003636), as well as myotonic dystrophy and fragile X syndrome (40.00% overlap, MCS=0).

**Table 1:** Disease pairs with comparable genetic overlaps have highly variable values for MCS

**Disease pair**	**Genetic overlap**	**MCS**
Parkinson Disease & Gaucher Disease	2.57%	0.064803
Fragile X Syndrome & ADHD	2.57%	0.002577
Obesity & Deafness	2.57%	0.000476
α-1 Antitrypsin Deficiency & Lung Neoplasms	2.58%	0.000996
Rheumatic Fever & Rheumatic Heart Disease	12.90%	0.170213
Hypertension & Breast Neoplasms	12.91%	0.003824
Cystic Fibrosis & Sarcoma	13.01%	0.000000
Ovarian Neoplasms & Breast Neoplasms	29.47%	0.169344
Multiple Sclerosis & Rheumatoid Arthritis	29.45%	0.027160
Spinal Muscular Atrophy & Fragile X Syndrome	29.63%	0.000000
Diabetes Mellitus & Obesity	35.70%	0.183136
Autism & ADHD	37.00%	0.003636
Myotonic Dystrophy & Fragile X Syndrome	40.00%	0.000000
Asthma & Hypersensitivity	74.71%	0.392971
Cryptococcosis & Poliomyelitis	75.00%	0.000000

**Figure 2:**
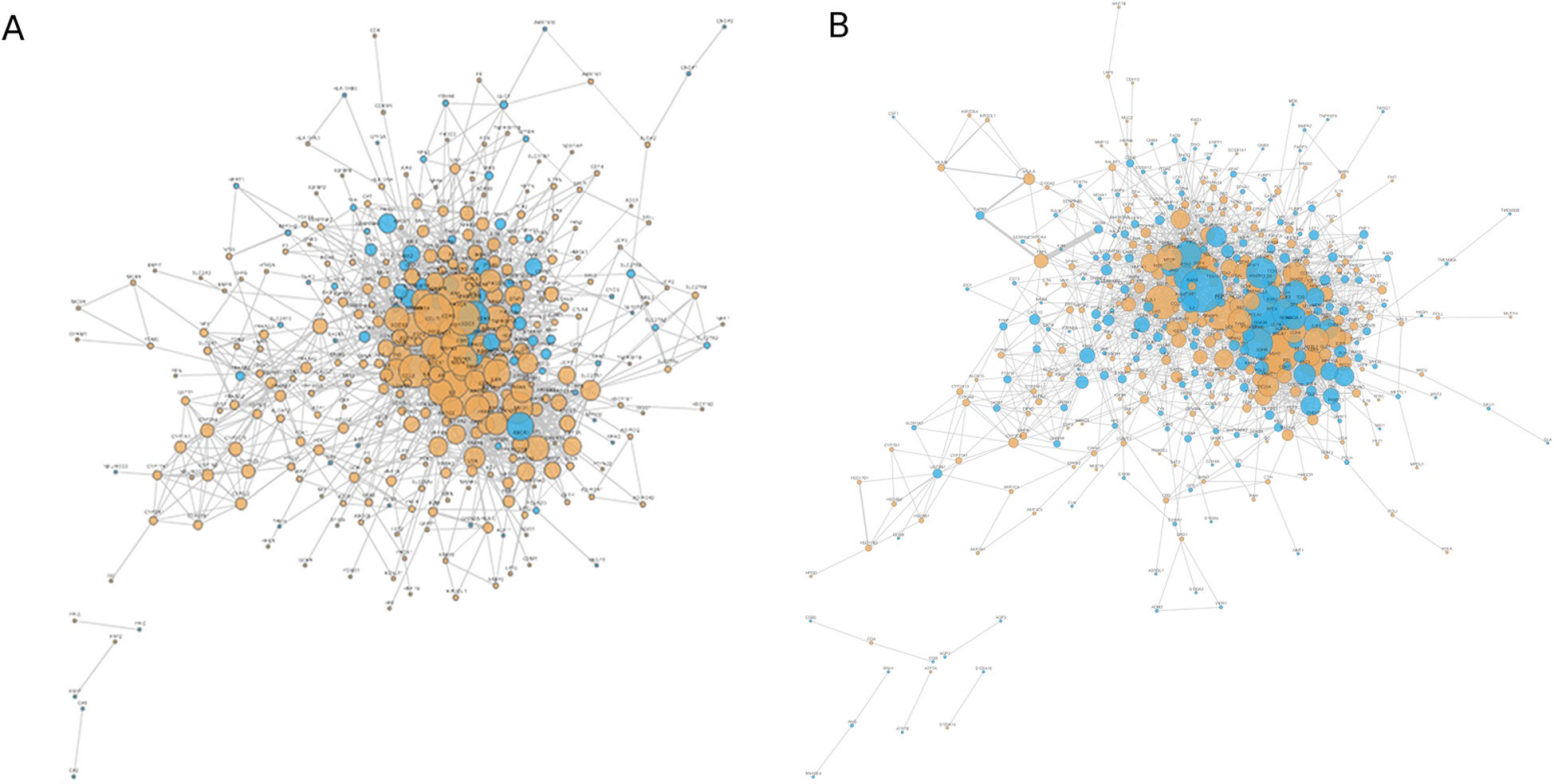
**The gene interaction networks of diabetes mellitus and obesity (A) as well as bladder neoplasms and lung neoplasms (B).** Orange nodes denote shared genes between the disorders. Even though the genetic overlap between the pairs is similar, the levels of mutual citation are significantly different.

The variability in MCS score appeared across all levels of genetic similarity. The disease pair of cryptococcosis, a potentially fatal fungal infection, and poliomyelitis, the viral infection commonly referred to as polio, had the highest genetic overlap (75.00%) of all the disease pairs studied. Despite its high genetic overlap, this pair was found to have an MCS of 0, indicating that no inter-disease collaboration has occurred throughout the 15 years covered in our analysis. However, asthma and hypersensitivity, which had the next highest genetic overlap (74.71%), returned the largest MCS value of any disease pair by a significant margin (MCS=0.392971). Figure 2 shows the gene interaction networks of diabetes mellitus and obesity (A) as well as bladder neoplasms and lung neoplasms (B). By comparing the two networks, we can see that both pairs share a considerable number of important genes, but despite the similar genetic relationships, the two pairs have largely different patterns and histories of mutual citation.

### Cross-pollination increases rate of discovery

**Figure 3:**
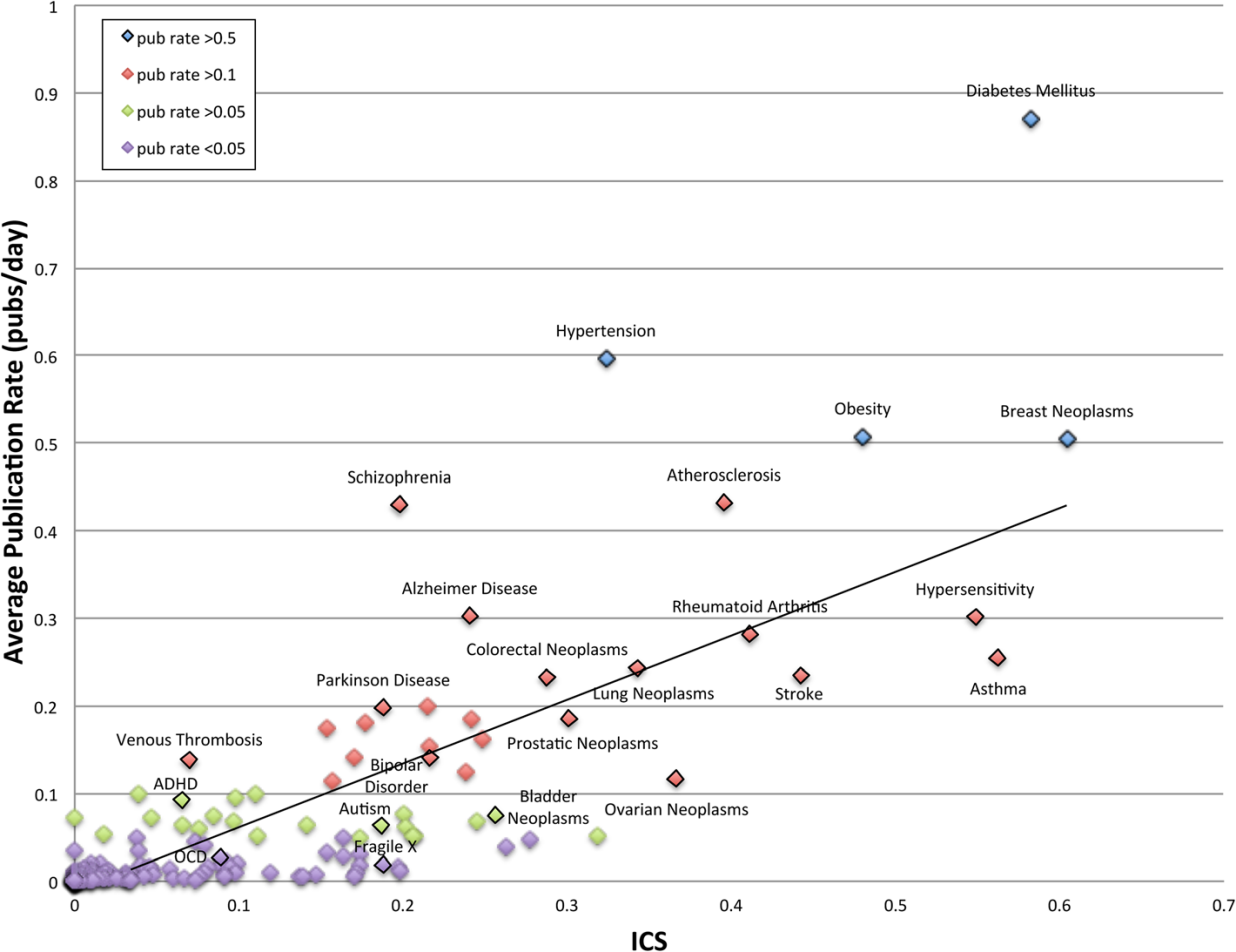
**Average rate of publication (publications/day) of disease-specific research fields with various levels of involvement in cooperative research (defined by the ICS).** From this figure it is clear that two genetically related diseases publish more often when cross-referencing their gene discoveries (diabetes mellitus and obesity), than when working independently (autism and ADHD) (r^2^=0.7931).

To weigh the implications of cooperative research efforts, we determined whether the presence or absence of cooperation predicted the impact of genetic discovery in general. Figure 3 illustrates a strong positive correlation (r^2^=0.7931) between the average rate of new discovery (publications/day) of a disease and its Individual Disease Cooperation Score (ICS), a measure of the total amount of cooperation occurring in an individual disease field (Additional file 3: Table S3). From this figure it is clear that a disease research field publishes more frequently when cross-referencing its gene discoveries with another genetically related disease than a research field that has worked independently despite high degrees of genetic overlap with other diseases. We found that disease fields that frequently referenced, or cross-pollinated, published genetic discoveries in other disease areas made novel genetic discoveries more rapidly than non-cooperative diseases, resulting in a complete genetic disease profile sooner. The observed correlation between the ICS and publication rate did not appear to be due to a “genetic bandwagon” effect in which disease fields follow the research trends of more active areas of research and discovery. When examining the balance in the number of citations from one disease to another for all disease pairs (limiting to those with 20 or more total citations), we found that a majority (61%) had near even numbers of co-citations, with 75% or more of the combined total evenly distributed between the two diseases in a pair (Additional file 4: Table S4).

The three disease fields that published most often were found to be diabetes mellitus, hypertension and obesity. Diabetes mellitus was found to have a publication rate of 0.87089 publications per day, translating to approximately 320 publications per year. Similarly, we calculated that the field of hypertension produces about 220 publications every year (pub rate=0.59701) and obesity, on average, releases 185 genetic publications per year (pub rate=0.05788). These diseases were also among the top fifteen most cooperative disease fields, as calculated by the ICS.

**Figure 4:**
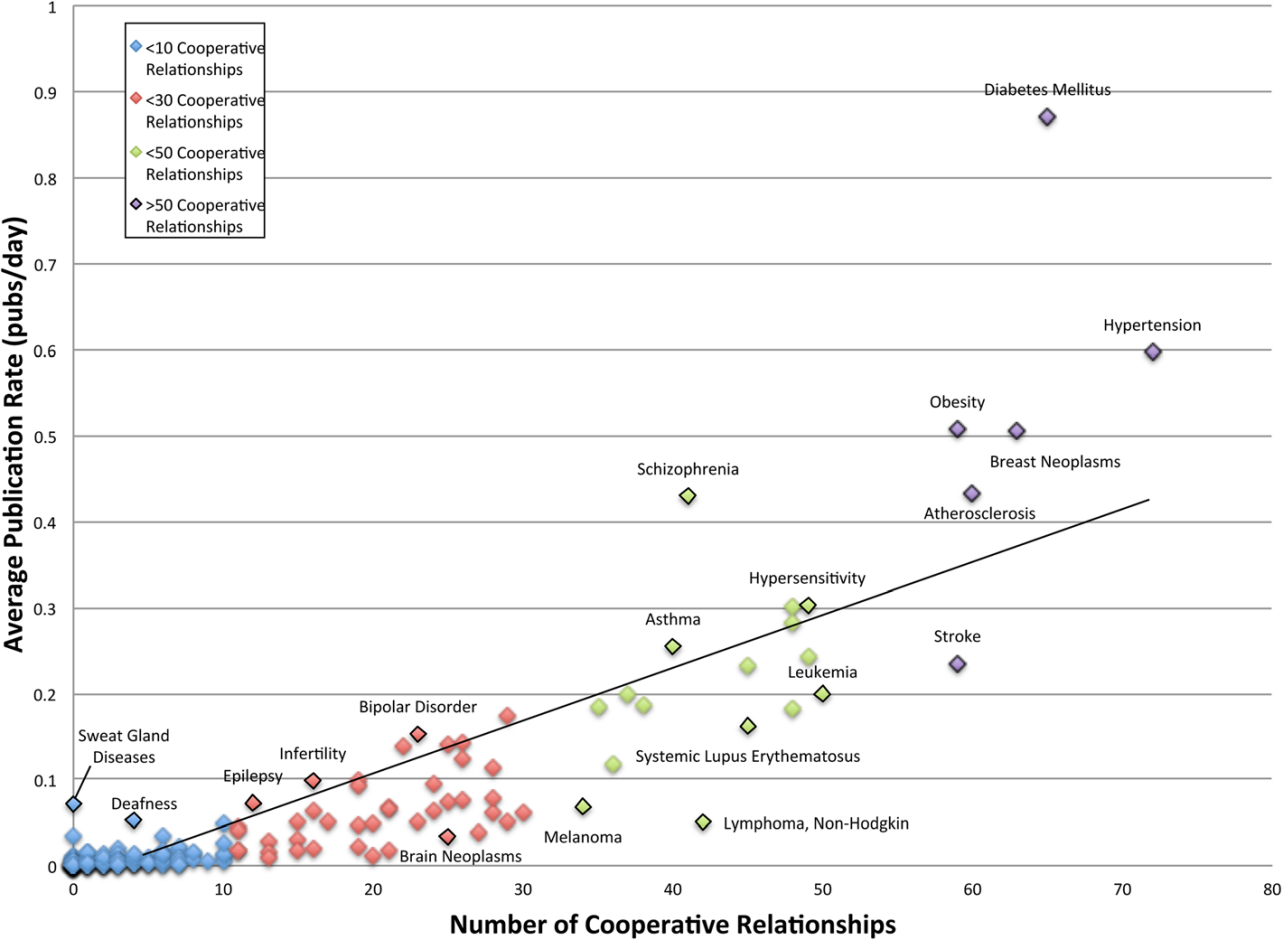
**Average rate of publication (publications/day) of disease-specific research fields with various numbers of cooperative partnerships.** From this figure it is clear that diseases publish more frequently when cooperating with a larger variety of genetically related diseases (r^2^=0.8585).

Furthermore, we determined that the number of cooperative partnerships was also highly correlated with the publication success of a given disease field (r^2^=0.8585), as depicted in Figure 4. Hypertension, diabetes mellitus and breast neoplasms were each found to have 72, 65, and 63 cooperative relationships, respectively. These were three of the top four most prolific fields we studied, in terms of publication rate. These data suggest that not only was the amount of cross-disease collaboration important for accelerating discovery, but that variety in collaborative partnerships also contributed to a faster rate of novel discovery.

The opposite was observed for disease fields that collaborated infrequently, or not at all, with related disease fields. These diseases were substantially slower at publishing novel genetic discoveries. For example, fragile X syndrome had a low publication rate of 0.018222 publications per day, or about six publications per year, as well as a poor ICS (ICS=0.1881). Likewise, fragile X syndrome was found to have only cited six of 70 related disease fields in its history of genetic publications. We also discovered that myotonic dystrophy failed to cite any other disease field in its genetic research, despite having high genetic overlap with over 40 diseases, including spinal muscular atrophy (38.10%) and fragile X syndrome (40.00%). As a consequence of this low level of inter-disease collaboration, myotonic dystrophy had a publication rate of 0.007194, translating to an average of only two or three genetic publications per year. This discovery rate is more than 100 times slower than that of diabetes mellitus, the leader in genetic publication, even though diabetes and obesity, the relationship that contributes most to diabetes mellitus’ ICS, share only 35.70% of their implicated genes, less than myotonic dystrophy shares with either spinal muscular atrophy or fragile X syndrome. Our data provides evidence that disease fields such as myotonic dystrophy and fragile X syndrome could greatly improve their publication rates by participating in more cooperative research.

## Discussion

By analyzing the publication histories of 193 genetic disorders over the last fifteen years, we found that cross-pollination of genetic discovery, or cross-disease research, is uncommon. Surprisingly, only 0.25% of all genetically related disease pairs studied exhibited inter-disease collaboration in greater than 5% of their total genetic research (MCS ≥0.05), and only about 10% of all disease pairs participated in cooperative research at any level (MCS >0.00), despite often large numbers of genes in common between the diseases.

We also found that when cross-pollination of genetic discovery does occur, the pace of discovery in the field is accelerated. Both the number of collaborative relationships -- the number of different diseases whose research was cited by a genetically similar disease -- and the Individual Disease Cooperation Score (ICS) were found to have significant positive correlations with publication rate. These results indicate that diseases that engage in collaborative research frequently and with a wide variety of related diseases tend to publish genetic discoveries more quickly than non-cooperative disease fields. Because the balance in co-citation among a majority of the disease pairs studied was high, we can conclude that the correlation between cross-pollination and accelerated discovery was due to a bidirectional sharing of research findings rather than a gene bandwagon effect and unidirectional tracking of “hot” disease fields.

Our results suggest that the field of human disease research has historically functioned mainly in a disconnected and single-disease focused manner rather than through collaborative multi-disease spanning effort. Part of this no doubt stems from our existing taxonomy of human disease and the importance of specialization, and potentially also to the funding priorities of federal funding agencies. However, our study provides evidence in support of the possible benefits of a shift towards more collaborative cross-disease research methods. Genetic discoveries made by cross-disease collaborations could provide insight into multi-disease indications for drugs, and since fields that participate in cross-disease collaborations tend to make discoveries faster, such a strategy would accelerate the beginning of clinical trials for these newly proposed therapies.

## Conclusions

The present study demonstrates the benefits of a cross-disease research model for genetic research in human diseases as they are currently defined. We found that a vast majority of genetically related diseases show no evidence for collaborative research practices over the last fifteen years. However, we observed that both the amount of cooperative research and the number of collaborative relationships in a particular disease field showed a strong positive correlation with an accelerated discovery rate in that disease field. These results suggest that cross-disease research will become increasingly more common and could accelerate the pace of discovery in the field as a whole, leading to faster understanding of the genetic roots of disease, faster development of multi-indicated drugs, and perhaps also leading to a new taxonomy of human disease that is informed by the expansive overlap in underlying genetic composition rather than by symptomatic traits.

## Abbreviations

MCS: Mutual citation score; ICS: Individual disease cooperation score; ADHD: Attention deficit disorder with hyperactivity.

## Competing interests

The authors declare that they have no competing interests.

## Authors’ contributions

DPW conceived of the project participated in algorithm design and analyses, and wrote the manuscript. MD participated in project design, coded the algorithms, conducted the analyses and wrote the manuscript. TN assisted in data acquisition and management and edited the manuscript. All authors read and approved the final manuscript.

## Pre-publication history

The pre-publication history for this paper can be accessed here:

http://www.biomedcentral.com/1471-2350/13/114/prepub

## Supplementary Material

Additional file 1 **Table S1.** Representation in PubMed Central for each disease field. Click here for file

Additional file 2 **Table S2.** MCS data and percent genetic overlap for each disease pair. Click here for file

Additional file 3 **Table S3.** ICS and cooperative partner data for each disease field. Click here for file

Additional file 4 **Table S4.** Citation balance computation for all disease pairs with 20 or more combined co-citations. Click here for file
